# Activation of regenerative pathways by exercise intervention in subjects with chronic obstructive pulmonary disease - a study protocol

**DOI:** 10.1186/s12931-025-03443-y

**Published:** 2025-12-12

**Authors:** Caroline Larsson, Linda Elowsson, Ellen Tufvesson, Anna Cederberg, Hugo Öhrneman, Bryan Falcones, Lisa Karlsson, Hamid Akbarshahi, Jaro Ankerst, Leif Bjermer, Andreas Palm, Jakob Löndahl, Andrei Malinovschi, Christer Janson, Margareta Emtner, Gunilla Westergen-Thorsson

**Affiliations:** 1 Human Movement: Health and rehabilitation, Department of Health Sciences, Lund University, Lund, Sweden; 2 Lung Biology, Department of Experimental Medical Science, Faculty of Medicine, Lund University, Lund, Sweden; 3 Respiratory Medicine, Allergology and Palliative Medicine, Department of Clinical Sciences Lund, Lund University, Skåne University Hospital , Lund, Sweden; 4 Division of Ergonomics and Aerosol Technology, Department of Design Sciences, Lund University, Lund, Sweden; 5 Department of Medical Sciences: Respiratory, Allergy and Sleep Research, Uppsala University, Akademiska sjukhuset, Uppsala, Sweden; 6 Department of Medical Sciences: Clinical Physiology, Uppsala University, Uppsala, Sweden

**Keywords:** Chronic obstructive pulmonary disease\, Exercise, Lung regeneration

## Abstract

**Introduction:**

Although Chronic Obstructive Pulmonary Disease (COPD) is generally considered a progressive condition with limited reversibility, recent data suggests potential for lung function improvement with exercise. Mechanistic insights into exercise-induced benefits remain limited, but in vitro models offer promise for understanding cellular and molecular changes. This study aims to elucidate the mechanisms behind lung tissue regeneration in COPD through adapted exercise training as well as validating advanced lung function tests, such as Impulse Oscillometry (IOS) and Airspace Dimension Assessment (AiDA) for improved detection of early physiological /structural changes and predicting clinical outcomes.

**Methods:**

This is the protocol of a multicenter, hypothesis-generating, single-arm study exploring the mechanisms for lung regeneration in subjects with COPD induced by exercise. The exercise protocol includes supervised and individually tailored moderate-intensity aerobic- and muscle strengthening exercise, performed three times per week for 12 weeks. Eighty sedentary adults with a clinically stable COPD will be recruited at two study sites. Included participants will be assessed at baseline and at 12 weeks by comprehensive pulmonary function testing (including spirometry, IOS, body plethysmography, diffusing capacity of the lung, single breath nitrogen wash-out, AiDA, questionnaires, physical capacity performance (including 6-minutes walking test (6MWT), one-minute sit-to-stand test, and cardiopulmonary exercise testing (CPET)), and collection of blood and urine samples and bronchoscopy. A mid-intervention assessment at week 6 will include medication use, health status, questionnaires, spirometry, and blood sampling.

**Discussion:**

Understanding the molecular and cellular activities related to lung function induced by exercise provides insights into repair pathways, challenges the notion of irreversible lung damage in COPD, and paves the way for improved management strategies with potential identification of biomarkers and pharmacological interventions.

**Trial registration:**

ClinicalTrials.gov ID NCT06335992 (Registration date 2024-03-28).

**Supplementary Information:**

The online version contains supplementary material available at 10.1186/s12931-025-03443-y.

## Background

Chronic obstructive pulmonary disease (COPD) is an increasing global health burden characterized by persistent airflow limitation and progressive destruction of lung tissue with no cure, leading to significant patient suffering and placing a considerable strain on healthcare systems [1]. The pathogenesis of COPD is primarily driven by long-term exposure to noxious agents—most notably tobacco smoke, but also air pollution, occupational hazards, genetic predispositions (e.g., alpha-1 antitrypsin deficiency), and early-life factors such as prematurity and recurrent respiratory infections. These exposures contribute to chronic epithelial injury, persistent inflammation, and remodeling of the extracellular matrix (ECM), ultimately leading to airflow limitation and clinical phenotypes such as chronic bronchitis and emphysema [2].

A hallmark of COPD is physical inactivity and poor physical capacity, both of which are associated with increased mortality, higher hospitalization rates, reduced health-related quality of life, and limited ability to perform daily activities. Pulmonary rehabilitation and structured exercise programs, such as aerobic and resistance training, have consistently been shown to improve functional performance and prolong survival [3–5]. As such, promoting physical activity and supporting patient self-management are key components of long-term COPD care. However, despite well-established clinical benefits, the underlying cellular and molecular responses to exercise in COPD remain poorly understood. Moreover, current pharmacological treatments primarily alleviate symptoms and reduce exacerbation frequency but do not halt or reverse the underlying pathophysiology. Gaining mechanistic insight into the biological responses to exercise is therefore essential for the development of disease-modifying and personalized therapeutic strategies.

COPD is a heterogeneous disease with substantial interindividual variability in underlying pathology. Central mechanisms include oxidative stress, chronic inflammation, and increased proteolytic activity. The inflammatory response may be dominated by neutrophils, eosinophils, or macrophages, often accompanied by elevated cytokines such as TNF-α, IL-1β, and IL-6. Structural changes including small airway remodeling, mucus hypersecretion, and alveolar destruction, vary in extent and contribute differently to clinical manifestations. A key driver of ECM degradation is the imbalance between matrix metalloproteinases (MMPs) and their endogenous inhibitors (TIMPs), resulting in excessive proteolysis of critical ECM components like elastin and collagen. This impairs alveolar integrity, reduces lung compliance, and contributes to air trapping and hyperinflation, hallmarks of progressive lung function decline [6, 7].

The general understanding has until now been that once COPD is established, it is irreversible and linked to a gradual loss of lung function over time. However, recent data from observational studies on COPD cohorts indicate that not all subjects decline in lung function, some even show improvements over time [8, 9]. The ability of adult human lung growth and alveolarization after pneumonectomy has been demonstrated in a case report study, where exercise and induced stretch were relevant contributions to the success [10]. Altogether, this indicates that the human lung has an inherent capacity to self-renewal.

This project therefore aims to uncover the mechanisms of exercise-induced lung regeneration, with a focus on improving disease monitoring and individualizing therapy.

To obtain deeper mechanistic insights into the in vivo molecular and cellular activities induced by physical exercise, we aim to do an extensive evaluation of subjects with a stable COPD before and after a 12-week intervention program. The exercise intervention is specifically tailored to stimulate the distal regions of the lung, areas most severely affected in COPD and essential for efficient gas exchange. Traditional lung function tests, such as spirometry, are often insufficient for detecting subtle changes in these peripheral lung compartments. Therefore, we aim to validate advanced, non-invasive techniques, including Impulse Oscillometry (IOS) [11] and Airspace Dimension Assessment (AiDA) [12], which offer improved sensitivity for assessing small airway and distal lung involvement. These methods may enhance the early detection of physiological changes and improve the prediction of clinical outcomes.

To complement the in vivo data, we will replicate exercise-related conditions in an in vitro bioreactor system developed in house, designed to mimic the lung’s microenvironment and mechanical forces [13]. This platform integrates cyclic stretch, mimicking in vivo breathing, with 3D cell culture systems using decellularized lung slices (DLS) and precision-cut lung slices (PCLS), enabling the study of tissue regeneration and extracellular matrix dynamics in both healthy and COPD-affected lung tissue. The findings will have implications for both basic science and clinical practice, by informing the design of more effective, targeted exercise protocols and enabling the monitoring of biological responses through accessible biomarkers in blood samples. By integrating physiological measurements with biomarker profiling and imaging, we aim to refine disease phenotyping, optimize diagnostics, and ultimately personalize COPD management. Ultimately the in vitro studies are aimed to find new targets for treatment of COPD that can act synergistically with physical exercise.

We hypothesize that individually adjusted physical exercise leads to; (1) reduction in harmful remodeling processes linked to oxidative stress, inflammation, and remodeling, and (2) activation of beneficial regenerative pathways. We also aim to identify and validate biomarkers that reflect these biological responses, both as indicators of lung regeneration and as potential therapeutic targets.

## Methods/Design

### Main objective

The main objective of this project is to identify mechanisms for lung regeneration in subjects with COPD induced by physical exercise. Our hypothesis is that adjusted physical exercise improves disease outcomes in these subjects by [1] decreasing remodeling processes linked to oxidative stress, and inflammatory and/or immunological pathways in the lung [2], inducing remodeling processes linked to healthy regeneration of lung tissue. Along the way, we also expect to identify (or validate) biomarkers mirroring systemic processes such as reduced inflammation and ongoing remodeling. These events may additionally act as potential targets for interventions.

### Specific aims

1. Study the effects of physical exercise on lung function parameters linked to structural properties e.g., elasticity, stiffness and distal air space dimensions.
2. Validate IOS and AiDA as sensitive and non-invasive tools for detecting changes in lung physiology, and determine their correlation with CT-derived emphysema measures.
3. Evaluate the effect of physical exercise intervention on lung physiology as measured by IOS and AiDA, and assess how these changes relate to changes in symptom burden and quality of life.
4. Evaluate changes in biomarkers before and after the exercise intervention related to regenerative processes, extracellular matrix turnover, stem/progenitor cell activity, and inflammatory patterns in lung tissue biopsies, blood- and urine samples. Correlate these changes with: (a) pulmonary function testing indices, (b) physical capacity, (c) health-related quality of life.
5. Explore the association between structural lung changes, as characterized by high-resolution computed tomography imaging (HRCT), and systemic biological responses, as measured by circulating and urinary biomarkers.
6. Investigate cellular behavior and tissue remodeling/regenerative responses in vitro using models derived from COPD subjects and evaluate how these processes are modulated by exercise-induced systemic changes.

### Trial design

**Fig. 1:**
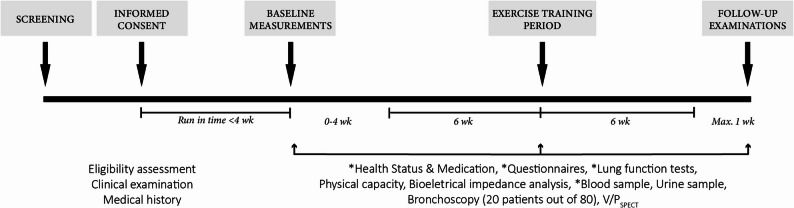
Overview of study design

This trial, “Tissue Regeneration and Exercise” (T-REX), is designed as a multi-center exploratory, hypothesis-generating single group trial exploring the mechanisms for lung regeneration in people with COPD induced by a 12-week exercise intervention. The project is divided into four parts; [1] basic baseline survey and testing; [2] a 12-week exercise intervention; [3] analysis phase; and [4] in vitro characterization. Figure 1 gives an overview of the study design.

This protocol is developed in compliance with the SPIRIT 2013 guidelines for reporting clinical trial protocols [14]. Figure 3 provides an overview of the SPIRIT schedule for enrolment, intervention, and assessment, and a SPIRIT checklist is included as Additional file 1. The intervention details are structured following the guidelines of the Template for Intervention Description and Replication (TIDieR) [15] (Additional file 2) and the Consensus on Exercise Reporting Template (CERT) [16] (Additional file 3).

### Study setting/participating centers

The study will be conducted at two study sites in Sweden: Lund and Uppsala. Principal investigators in Lund are Gunilla Westergren-Thorsson and Leif Bjermer and in Uppsala Margareta Emtner and Christer Janson. The following centers will be involved in the project.

1. Lund University, Lund, Sweden.
2. Region Skane, Lund, Sweden.
3. Uppsala University, Uppsala, Sweden.
4. Region Uppsala, Uppsala, Sweden.

Enrolment began in 2022 at both sites and is scheduled to run until the recruitment of eligible COPD subjects has been finalized. The first round of recruitment is expected to be completed end of 2025.

### Eligibility criteria

#### Inclusion criteria

Participants will be adults with a diagnosis of COPD. To be eligible for participation in the study, participants must have:

1. Forced expiratory volume in one second (FEV_1_)/Forced vital capacity (FVC) < 0.7.
2. Postbronchodilator FEV_1_ of 30–85% of predicted value.
3. Optimal medical treatment according to GOLD and the Swedish National Guidelines.
4. Former smokers, ≥ 10 pack-years, smoking history, but had to be an ex-smoker for ≥ 1 year.
5. Free from exacerbations in the last 6 weeks.
6. Age: 50–85 years.
7. BMI: 18–37 kg/m^2^.

#### Exclusion criteria

Individuals may not enter the study if any of the following apply:

1. Regular participation in exercise sessions (≥ 2 times/week).
2. More than one exacerbation during the 12 months prior to study entry.
3. Long-term oxygen treatment (LTOT).
4. Oral corticosteroids or biological treatment.
5. Desaturation to ≤ 87% at the screening six-minute walk test (6MWT).
6. Alcohol or drug abuse.
7. Unstable cardiovascular or pulmonary disease.
8. Any physical or mental condition, including comorbidities that, at the discretion of the investigator, are considered likely to interfere with safe participation in the exercise program or influence the study outcomes.

### Intervention

#### Intervention description

The 12-week exercise intervention will consist of moderate intensity aerobic and strength training twice per week as “supervised training on site” and once per week as “training at home”. All exercise sessions on site will be conducted in groups of 3–8 people in a public fitness setting and supervised by a knowledgeable and experienced physiotherapist. Supervision of exercise will include providing motivation and feedback and/or modifying exercises as appropriate/required by, for example, changing the resistance, pace or number of repetitions. The physiotherapists will adhere to a single training protocol to ensure standardized delivery of the training across centers, and regular meetings during the trial will be used to check that the procedures are carried out in the same way at both sites.

The supervised training sessions on site will include.

1. Aerobic exercise for 33 min on a cycle ergometer at a moderate intensity (≥ 60% Wpeak), individually tailored to achieve a perceived exertion level of 3–6 on the Borg CR-10 scale [1]: After 3 min of warm-up at a low intensity, the exercise will be performed in intervals, 2 min at a moderate intensity, 50–60 revolutions per minute (rpm), 1 min at low intensity, 2 min at a moderate intensity etcetera, in total, 10 intervals at moderate intensity. The exercise intensity will be based on symptom limitation and tailored to the individual needs and limitations of the subject. As one exclusion criterion is oxygen saturation (SpO_2_) ≤ 87% at the screening 6MWT, we do not expect that anyone will desaturate during the training. However, if SpO_2_ decreases < 85% during cycling the training will be stopped and commence when SpO_2_ > 90% [17]. If this happens more than once the patient will be excluded.
2. Strength training for 30 min, involving seven exercises targeting major upper and lower body muscle groups. Each exercise comprises three sets with 8–12 repetitions. For the muscle strengthening exercise equipment such as graded elastic bands and handheld dumbbells will be used. The exercise load will be determined by the participant’s ability to complete 10 repetitions for a given exercise and progression will be achieved by increasing the number of sets, weight or elastic band resistance, as guided by the physiotherapist.

The training at home will include aerobic and strength training for about 60 min. To reduce variability and support adherence in the home training, participants will receive detailed instructions, an exercise diary, and the home-training will be followed-up by the physiotherapist on a regular basis.

1. The aerobic training at home will include individually adapted aerobic training such as walking continuously or in intervals, cycling or swimming.
2. The strength training will include the same exercises, sets and repetitions as in the “on-site training”, but can be changed according to the choice, need and ability of each subject.

All participants will receive a handbook containing information about the benefits of physical training, practical tips on exercise, an exercise diary for home training where they can plan and track their progress, and contact information with the physiotherapist.

### Criteria for discontinuing or modifying interventions

If participants, due to illness or other absences, are unable to adhere to the training protocol, the training period will be extended. In cases where participants experience illness or absence during the intervention, the training period will be extended to ensure that each participant completes a full six-week training program at the end of the intervention.

Participants have to be in a stable state of their COPD disease as the appearance of increased inflammatory markers may be confounding factors in the analysis of the results.

### Strategies to improve adherence to intervention

To enhance adherence the exercise intervention will be tailored to each individual’s needs and current condition. Continuous support and follow up will take part during the training sessions, and all participants will have access to daily support from the trainers via phone or text messages. Participants will be encouraged to always contact the physiotherapist in case of illness or absence and a plan for continuation will be planned and modified when needed.

### Concomitant and post-trial care

Participants will be allowed to continue their regular medication. However, any changes in medication will not be permitted. If an increase in medication for the lung disease is required, it will be considered an exacerbation, resulting in an extension of the training period. The participants are not allowed to start with any other exercise intervention during the time period of the training intervention. All participants will have a patient insurance. Physician contact will be available during the study.

### Patient and public involvement

Patients and/or the public were not involved in the development of the research question, study design, choice of outcome measures, or recruitment process. This decision was based on the exploratory and mechanistic nature of the study, which primarily focuses on physiological, molecular, and imaging outcomes. However, participants are actively engaged in the intervention phase through their involvement in the tailored exercise program.

### Study outcomes

The primary outcome is to validate the effect in protein profiles after exercise intervention in lung tissue biopsies, blood- and urine samples using proteomic analyses such as multi-plex ELISA, OLINK, and mass spectrometry. Primary factors include; matrix turnover/remodelling proteins: MMPs, TIMPs, MMP-degraded collagen peptides, desmosine, periostin, decorin; growthfactors: VEGF, HGF, PDGF, FGF-2, angiopoietin; inflammatory/immune markers: TNF-a IL-6, IL1-b, IL-8, IL-10, IL-17, MCP-1, CXCL10, CXCl13.

### Secondary outcomes

The secondary outcomes are to validate the effect of training to:

- Identify responders and non-responders based on measurements of vital lung parameters (e.g., IOS, FEV_1_, DLCO, AiDA) and physical capacity (6MWT, sit-to-stand test) after exercise intervention.
- Identify changes in biomarkers related to regenerative processes, matrix turnover, stem/progenitor cell activity, and inflammatory patterns in lung tissue biopsies, blood- and urine samples, and correlate these changes with vital lung parameters (e.g., IOS, FEV_1_, DLCO, AiDA) and measures of physical capacity (e.g., 6MWT, sit-to-stand test).
- To validate the effect of the exercise intervention on vital lung parameters based on established and novel lung function measurements e.g., IOS, spirometry, DLCO, AiDA, Cardiopulmonary exercise testing.
- To validate the effect of the exercise intervention on the physical capacity based on established measurements 6-MWT, sit-to-stand test.

### Other secondary outcomes

Other secondary outcomes are to validate:

- Improved health-related quality of life parameters after exercise intervention.
- Improved exercise capacity.
- Validate if gender and age are confounding factors for the effect of the exercise intervention.

### Exploratory outcomes

Anticipated exploratory outcomes are to:

- Validate AiDA as a better non-invasive method to establish degree of emphysema in diagnosis of COPD.
- Validate regenerative processes, matrix turnover, stem/progenitor cell activity in the in vitro models that recapitulate the microenvironment of distal lung tissue from COPD subjects using DLS and PCLS in our developed physiomimetic stretch system [13] and assess how these processes are modulated by exercise.
- Identify key signaling pathways involved in extracellular matrix turnover, stem/progenitor cell activity, and inflammatory regulation within the in vitro COPD model, and determine how these pathways interact synergistically with mechanosensing signaling cascades activated by exercise-mimicking mechanical stimuli.

### Screening tools

Screening for problematic alcohol use will be conducted using the Cut-down, Annoyed, Guilty, and Eye-opener (CAGE) questionnaire [18] to identify participants at risk of dropout and to support completion of the training period. The questionnaire consists of seven questions, which can be answered with “Yes” or “No.” Two “Yes” responses to questions 2–7 suggest alcohol dependence.

Screening for adequate activity levels will assess participants’ physical activity over a typical week using a questionnaire [19, 20]. Structured exercise (Question 1) will cover activity type, duration, and intensity, while everyday activities (Question 2) will include daily routines, walking habits, and weekly activity patterns. Data from these questions will be used to calculate total activity minutes (exercise minutes ×2 + everyday activity minutes ×1). Participants engaging in organized and regular exercise ≥ 2 times per week will be excluded.

Subjects will be interviewed about medical history, medications, smoking status, exacerbation history and infection status.

### Study procedures

At first visit, all subjects will initially go through a clinical examination including electrocardiogram. Before first measurement the subjects will inhale 400 µg short acting beta-2 agonist (salbutamol, Buventol^®^ Easyhaler) using a valve spacer device. All testing will be performed at least 15 min thereafter in the following order: fractional exhaled NO (FeNO), 6MWT, IOS, Spirometry, Body plethysmography, DLCO, SBW, 1-minute sit-to-stand test, 6MWT no 2 (at baseline visit), Cardiopulmonary exercise testing (CPET) - which might be done at a separate visit. Subjects will be asked to rest when needed between tests, and specifically at least 10–15 min after 6MWT and sit-to-stand test. If the subject fulfills any of the exclusion criteria, no further testing will be performed.

Blood and urine samples will be taken, and questionnaires will be filled in during resting times. Aida will be done on a separate visit.

### Clinical characteristics

A routine physical examination by a physician will be performed. Medical history, medications, smoking status, allergy status, exacerbation history, infection status, blood pressure, heart rate and SpO_2_, and electrocardiogram (ECG) in supine position will be recorded.

Bioimpedance.

To assess body composition, bioimpedance is used to estimated body fat and lean muscle mass.

### Questionnaires

COPD specific questionnaires will be used to identify symptom burden and health-related quality of life (HRQoL). The COPD Assessment test (CAT) is an 8-item, validated questionnaire, with good discriminant properties. The scoring ranges from 0 to 5 points for each question, i.e., resulting in a total score of 0–40, and with a lower the score indicating better health status. A change of 2 units has been used as a minimal clinically important difference (MCID) [21].

Clinical COPD questionnaire (CCQ) is a HRQoL questionnaire. It includes 10 questions about the participants well-being the last week scoring from 0 (= never) to 6 (= almost all the time) a total score of (0–60)/10, and with a lower the score indicating better health status [22]. A change of 0.4 units has been used as a MCID [23]. Modified Medical Research Council (mMRC) scale measures dyspnea. It measures dyspnea in 5 steps (0–4) with 0 being the least and 4 being the most dyspnea [24].

### Exercise tests

#### Six-minute walk test (6MWT)

The six-minute walk test (6MWT), a sub-maximal exercise test, is used to assess aerobic capacity and endurance [24]. In the 6MWT, the subjects are asked to walk, in a 30-meter distance, as far as possible during six minutes. The following parameters will be observed: walking distance, SpO_2_, heart rate, perception of dyspnea and leg fatigue every two minutes during the test [25]. The 6MWT will be performed twice at the baseline visit, and the results from the best performance (= longest distance) is used. The MCID is 30 m [25, 26].

#### One-minute sit-to-stand test

The one-minute sit-to-stand tests measures leg muscle strength [27]. The subject starts in a sitting position, and during one minute stands up from a chair (height 46–48 cm) and sits down again as many times as possible. The number of times to stand up is the main outcome. SpO_2_, heart rate, perception of dyspnea and leg fatigue before and after the test will be assessed [27]. The MCID is three repetitions [27].

#### Cardiopulmonary exercise testing (CPET)

Cardiopulmonary exercise testing (CPET) is a method for standardized testing of exercise capacity and will be performed during cycling. In short, during the CPET numerous components are observed: electrocardiogram changes, blood pressure readings, heart rate monitoring, gas analysis breath-by-breath (such as oxygen uptake [VO_2_] and carbon dioxide elimination [VCO_2_]), minute ventilation (V_E_) and subjective ratings of perceived exertion during every second minute during the test. There is an initial phase of breathing in the mask for 3 min, to stabilize the equipment, followed by a pedaling for 3 min without any workload. Thereafter women will start on a work load of 20 W and men at 30 W followed by a ramp increase in workload (10 W/minute) until exhaustion. We aim for a duration of 8–10 min and a minimum duration of exercise of 6 min.

The CPET is performed using an electronically braked cycle ergometer and breath-by-breath gas analysis equipment (Vyntus CPX Metabolic Cart, Vyaire Medical, Mettawa, IL, United States) for gas analysis. The data analysis is performed using SentrySuite software version 3.20 (Vyaire Medical, Höchberg, Germany) for CPET parameters. Reference values for VO_2_peak are from Gläser 2010 [28]. MCID for VO_2_ is established to 5–10% [29].

#### Fractional exhaled NO (FeNO)

Fractional exhaled NO (FeNO) is measured using an Ecomedics, CLD88 (Dürnten, Switzerland). The test starts with a maximum exhalation, then an inhalation of NO depleted air from the mouthpiece, and thereafter an exhalation at a flow of 50 ml/s. The device uses a built-in flow restrictor to maintain the exhalation flow and provides real-time visual feedback to ensure correct performance. The mean of three adequate measurements will be recorded [30].

#### Lung function tests

##### Spirometry

Flow volume spirometry will be performed to measure forced expiratory volume in 1 s (FEV_1_) and forced vital capacity (FVC). FEV_1_/FVC is calculated as a measure of expiratory flow limitation. Spirometry is performed according to ERS guidelines [31] (using a Jaeger MasterScope; Hoechberg, Germany) in an upright position with a nose clip. The best of three adequate measurements will be recorded. As a Swedish population will be investigated the reference equations from Hedenström et al. [32, 33] will be used to calculate % of predicted values.

### Impulse oscillometry (IOS)

IOS provides information about the mechanical properties of large and small airways (Jaeger MasterScreen IOSHoechberg, Germany). Resistance is estimated at 5 Hz (R_5_, reflecting total resistance) and at 20 Hz (R_20_, reflecting central resistance), while R_5_-R_20_ is subsequently calculated and reflects peripheral resistance. Airway reactance (elastance) of the lung is estimated at 5 Hz (X_5_), which during small airway obstruction gives a more negative value, while the resonant frequency (F_res_) increases together with the area under the reactance curve (AX), which is measured from 5 Hz to F_res_. The test is performed in an upright position and the subjects hold their hand palms on their cheeks to stabilize the upper shunt [34]. The mean of three accurate measurements of 30 s is recorded.

### Body plethysmography

Body plethysmography is used (MasterScreen Body, Erich Jaeger GmbH), to measure static lung volumes according to European Respiratory Society (ERS) standards [35], i.e. total lung capacity (TLC) and residual volume (RV), and the ratio RV/TLC is calculated as a measure of air trapping. In addition, total (R_tot_), inspiratory (R_in_) and expiratory (R_ex_) resistance are measured. The mean of three accurate measurements is recorded.

### Diffusing capacity of the lung

Diffusing capacity of the lung for carbon monoxide (CO) (DL_CO_), the rate constant for uptake of CO (K_CO_) and alveolar volume (VA) will be measured/calculated using the MasterScreen Pulmonary Function Test (PFT) equipment (Erich Jaeger GmbH). A single-breath carbon monoxide diffusion test is performed by a breath-hold for 10 s, according to ATS/ERS standards [36], in a sitting position and wearing a nose clip. CO and methane are used as tracer gases. The mean of three accurate measurements is recorded.

### Single breath nitrogen wash-out

Single breath nitrogen wash-out measures ventilation heterogeneity in the peripheral airways by analysing the slope of the alveolar phase of a breath. The maneuver is performed by a maximum exhalation, maximum inhalation and thereafter maximum exhalation again using an Exhalyzer D (Eco Medics, Dürnten, Switzerland). The test is performed in a sitting position and wearing a nose clip. The mean of the phase III slope (SIII) of three accurate measurements is recorded.

### Airspace dimension assessment (AiDA)

AiDA is a novel method used to measure the dimensions of the distal airspaces by analyzing inhaled and exhaled concentrations of 50 nm aerosol particles. The deposition fraction over several breath-holds is used to calculate an average distal airspace radius (r_AiDA_) and a zero-second recovery (R_0_), which is assumed to be determined by geometrical properties of the peripheral lung, such as airway heterogeneity [12, 37]. The breathing manoeuvre is similar to that used for single-breath D_LCO_, with the subject breathing into a mouthpiece while wearing a nose clip. The subjects breathe particle-free air for approximately 30 s, after which air is exhaled to residual volume and then aerosol particles are inhaled to total lung capacity. Thereafter, the subjects hold their breath for a few seconds, and then exhale. The aerosol concentration is measured from exhaled air after washout of dead space. The manoeuvre is repeated with breath-holds of 5, 7 and 10 s, with at least two successful replicates of each, for a total of at least six measurements.

The technical principle of AiDA and how the distal airspace radius, *r*_AiDA_ and the R_0_ have been described in more detail elsewhere [37]. Prior to further analysis, the adherence to the breathing manoeuvre and the stability of the particle generation will be evaluated for each measurement, and low-quality measurements will be excluded.

### High resolution computer tomography (HRCT)

High resolution computer tomography (HRCT) will be performed according to clinical routine. Emphysema will be classified as centrilobular, panlobular or paraseptal. Additional visual features to be evaluated include airway wall thickening, small airway disease, tracheal and interstitial abnormalities, pulmonary arterial enlargement and bronchiectasis [38]. Grading of emphysema will be done as volume with low and high attenuation density of the respective lung. A Specialist in Clinical physiology will quantify volumes with low and high attenuation density in the lung tissue from the HRCT pictures, by means of an objective imaging density measurement.

### Sample collection

Inflammatory biomarkers, regeneration components and cellular profiles will be investigated in blood, urine and BAL samples.

### Blood samples

All blood samples are collected before 13.00 o’clock to avoid any effect of diurnal variation. Blood will be drawn in EDTA tubes for differential cell count (performed at the hospital routine laboratory), for plasma (collecting the supernatant after 10 min centrifugation in 2000 x g) and for buffy coat (collecting the white blood cell layer after the centrifugation). In addition, serum will be sampled (after 30 min incubation to enable coagulation and collecting the supernatant after 10 min centrifugation in 2000 x g). Samples will be frozen in −80 °C until further analyses. Levels of hemoglobin and alpha-1 antitrypsin will be measured according to clinical routine.

### Urine samples

Urine will be collected and frozen in −80 °C until further analyses.

### Bronchoscopy samples

Bronchoscopy is performed in accordance with clinical routine to obtain bronchial biopsies and bronchoalveolar lavage (BAL) samples. Six biopsies are collected from the left lower lobe using the cryobiopsy technique, with a freezing time of four seconds. BAL is carried out by instilling two aliquots of 50 mL of body-temperature isotonic saline into the lingula or middle lobe, followed by gentle aspiration [39, 40].

### Participant timeline

Participants will first be contacted by phone or mail for an initial screening. Before the intervention begins, they will undergo a comprehensive baseline assessment (one to three visits). Following this, participants will engage in a 12-week supervised exercise intervention, with data collection scheduled at mid-intervention (after 6 weeks, one visit) and post-intervention (after 12 weeks, one to three visits).

The baseline and post-intervention assessments will include medication use and health status, clinical examination by a physician, collection of participant data, questionnaires, physical capacity tests, comprehensive pulmonary function assessments, and sample collection.

The mid-intervention assessment will cover medication use, health status, questionnaires, spirometry, and blood sampling.

**Fig. 2:**
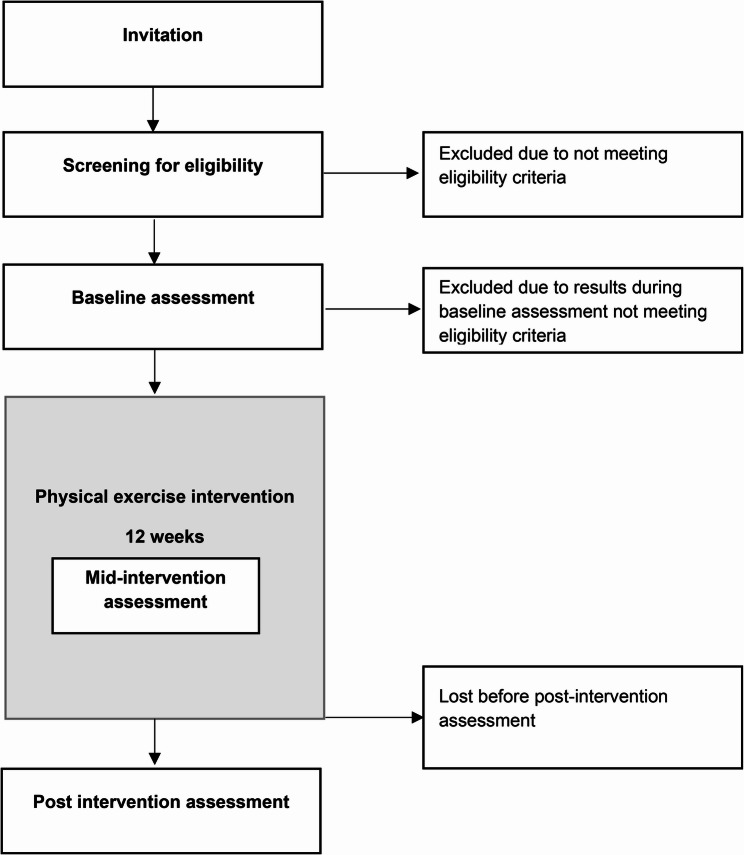
Flow diagram of participant recruitment

**Fig. 3:**
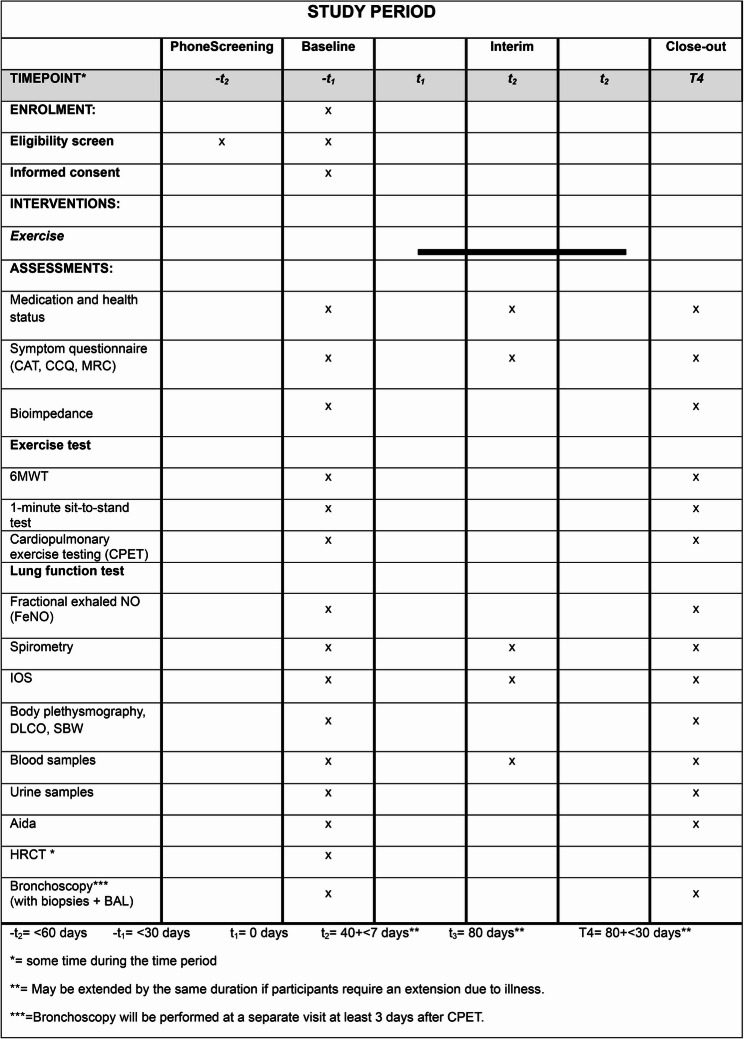
SPIRIT diagram illustrating the schedule of enrolment, interventions and assessments

Participant recruitment is summarized in a flow diagram (Fig. 2), and the schedule of enrolment, intervention, and assessment are presented in the SPIRIT diagram (Fig. 3).

### Order of assessments and procedures during study visits

The order of assessments and procedures during each study visit has been standardised to ensure consistency and minimise participant burden. At each visit, procedures will be conducted in the following sequence: Fractional exhaled NO (FeNO), 6MWT, IOS, Spirometry, Body plethysmography, DLCO, SBW, sit-to-stand test, 6MWT no 2 (at baseline visit), Cardiopulmonary exercise testing (CPET), which might be done at a separate visit. Subjects will be asked to rest when needed between tests, and specifically 10–15 min after 6MWT and sit-to-stand test. Blood and urine samples will be taken and questionnaires will be filled in during resting times. Aida will be done at a separate visit.

### Sample size

This is an exploratory study designed to include 80 subjects with clinically stable COPD (assuming a 10–20% drop-out rate) to accommodate various degrees of biological variations in the different exploratory outcome measures. Of these, 20 will also undergo bronchoscopy. Findings that do not reach statistical significance in a sample size of approximately 80 subjects are likely to have limited clinical relevance.

### Recruitment procedure

Participants diagnosed with COPD will continuously be recruited from primary and specialist care in and around Lund/Malmö and Uppsala regions in Sweden. We will also advertise through, for example, social media and patient organizations (e.g., the patient associations) as well as advertising in local and national newspapers. Telephone screening will be used to identify and invite eligible participants for inclusion in the study.

### Trial status

The study is currently recruiting participants.

### Plans for assessment and collection of outcomes

The assessment and collection of data will be conducted by specially trained research nurses with extensive experience of performing the study related assessments and collecting data from participants. All testing is done according to standardized procedures and with validated protocols. All equipment fulfils standardized requirements. The basic baseline assessments will be carried out before (initial visit) and after the completion of the training program (follow-up visit post-training program), as well as a less comprehensive examination after halfway through the training program.

### Plans to promote participant retention and complete follow-up

Plans to promote participant retention will include frequent contact with study team including calls for symptom checks, and reminder calls prior to scheduled study visits. We will aim to collect as much outcome data as possible in participants who discontinue or deviate from the protocol, including detailed records of reasons for discontinuation.

### Data management

We will use the Research Electronic Data Capture system (REDCap), which is offered to affiliated research group leaders at the Faculty of Medicine at both Lund and Uppsala universities to collect, store and report clinical research data in a secure manner. To ensure data accuracy and confidentiality, all data will be initially recorded on paper Case Report Forms (CRFs), then verified and entered into REDCap by data management personnel. Consenting participants will be assigned a unique sequential participant identification code which will be used to pseudonymize all data. This study ID number will be used in all data collection and analyses. All personal data will be processed in accordance with the EU General Data Protection Regulation (GDPR). Code list, paper CRF and signed informed consents will be stored in a locked cabinet in the respective research centers.

### Statistical methods

After the last participant has completed all assessments, additional monitoring and data quality checks will be completed. Analyses will be performed by the investigators supported by a biostatistician when needed. All statistical tests will be conducted as two-sided, with the alpha level set at 0.05. Baseline variables and descriptive statistics with continuous data will be presented with mean ± standard deviation (SD) or median with interquartile range (IQR) as appropriate. Ordinal variables will be presented as median (IQR), and categorical variables will be presented as n (%).

Continuous data with normative values (for example lung function values) will be presented both as absolute values and as % of predicted normal values.

The primary outcome is the change from baseline to post intervention, and analyses will be performed with paired statistical testing. Analyses of both absolute change (delta values) and % change will be performed. Age, sex, pack-years and site are potential confounding factors which might be taken into account as co-variates.

### Data monitoring

Participants will be monitored by physiotherapists during training sessions to identify signals that would require interruption of the session or exclusion from the trial. All lung function testing and CPET will be supervised by experienced nurses or biomedical technicians, and all data will be interpreted by specialists. A data monitoring committee is composed of the responsible researchers, physicians and physiotherapists, and will discuss adjustments to individual tasks. All data analysis is independent of a sponsor.

Any significant protocol modifications, including changes to the study aims, primary outcomes, design, sample size, or statistical analysis, will be reported to the Ethics Committee that authorized the trial. Additionally, these modifications will be updated in the ClinicalTrials.gov registry and transparently documented in trial reports and publications.

### Adverse event reporting and harms

Adverse events (AEs) will be documented in the CRFs or training protocols and assessed by researchers for causality related to the tests and/or intervention. The nature, timing, duration, severity, and outcome of AEs will be recorded and evaluated. All AEs will be promptly addressed and reported to the study steering committee. If necessary, appropriate actions will be taken. Any costs related to adverse events will be covered by the Swedish Patient Injury Insurance and no additional expenses will be incurred by participants.

### Auditing

There is no other pre-planned auditing of the trial. Authorities might do an audit of the study. The funder requires scientific and financial project reviews.

### Dissemination plans

The results from this trial will be disseminated regardless of the magnitude or direction of any possible effect. The results of the trial will be communicated to participants, healthcare professionals, the public, and other relevant groups through various channels, including peer-reviewed publications, presentation at scientific conferences as well as in newspapers and social media and through patient organizations. We will ensure that participants are informed of the study’s outcomes through individual updates at the post intervention session.

Authorship eligibility will be based on substantial contributions to the design, conduct, data analysis, or interpretation of the study results. All authors will be required to meet these criteria in accordance with standard academic guidelines.

Access to data is based on the established collaboration agreements including material and data transfer agreements. Efforts will be made to ensure transparency and allow for independent verification and analysis of the study’s findings.

## Discussion

In this study, we aim to identify mechanisms that activate regeneration of lost lung tissue to restore lung function in COPD, i.e. stimulating health- and not disease factors. Our hypothesis is that adjusted exercise training improves disease outcomes in subjects with COPD by inducing inherent regenerative processes or block further remodeling in the lung. By gaining deeper insight into the molecular and cellular responses triggered by exercise training, we aim to identify the key repair pathways involved, thus paving the way for potential enhancement through targeted pharmacological interventions. Furthermore, we expect to identify biomarkers mirroring these processes systemically to enhance our ability to monitor disease activity, personalize therapy, and stratify subjects into responders and non-responders. Moreover, we aim to validate IOS and AiDA as sensitive, non-invasive tools for detecting early physiological changes and disease progression, potentially more accurately than conventional spirometry, particularly for assessing small airway and distal lung pathology. We foresee that this project may create a foundation for other patient groups, where an early intervention with adjusted exercise training may have a substantial impact on recovery time and quality of life.

## Supplementary Information

Supplementary material 1.

Supplementary material 2.

Supplementary material 3.

## Data Availability

Involved researchers will have access to anonymized study data. Access for external parts to the datasets used and/or analysed during the current study will available be available in collaboration with responsible study investigator on reasonable request.

## Abbreviations

COPD: Chronic obstructive pulmonary disease
rAiDA: Average distal airspace radius
R0: Zero-second recovery
BAL: Bronchoalveolar lavage
REDCap: Research Electronic Data Capture
CRFs: Case Report Forms
SD: Standard deviation
IQR: With interquartile range
AE: Adverse events
HRCT: High-resolution computed tomography
VO2: Oxygen uptake
VCO2: Carbon dioxide elimination
R5: Total resistance
R20: Central resistance
R5-R20: Peripheral resistance
X5: Estimated airway reactance (elastance) at 5 Hz
Fres: Resonant frequency
AX: Reactance curve, measured from 5 Hz to Fres
